# Natalizumab Pharmacokinetics and -Dynamics and Serum Neurofilament in Patients With Multiple Sclerosis

**DOI:** 10.3389/fneur.2021.650530

**Published:** 2021-04-14

**Authors:** Undine Proschmann, Hernan Inojosa, Katja Akgün, Tjalf Ziemssen

**Affiliations:** Department of Neurology, Multiple Sclerosis Center, Center of Clinical Neuroscience, University Hospital Carl Gustav Carus, Dresden University of Technology, Dresden, Germany

**Keywords:** multiple sclerosis, natalizumab (TYSABRI), natalizumab concentration, neurofilament light (NFL) chain, recurrence of disease activity, cessation of natalizumab, alpha-4 integrin expression, alpha-4 integrin receptor saturation

## Abstract

**Background:** Natalizumab (NAT) is a high-efficacy treatment for relapsing remitting multiple sclerosis (RRMS). However, it is associated with an increased risk of progressive multifocal leukoencephalopathy that sometimes requires treatment cessation with a risk of returning disease activity. The aim of this study was to characterize the pharmacokinetics and -dynamics as well as neurodestruction marker serum neurofilament light chain (sNfL) in patients with RRMS and secondary progressive MS (SPMS) stopping NAT in correlation to clinical data.

**Methods:** In this study, 50 RRMS and 9 SPMS patients after NAT cessation were included. Five RRMS patients on NAT treatment holiday were evaluated. Clinical and radiological disease activity were systemically assessed by frequent exams after NAT stop. Free NAT concentration, cell bound NAT, α4-integrin expression and α4-integrin-receptor saturation as well as immune cell frequencies were measured for up to 4 months after NAT withdrawal. Additionally, sNfL levels were observed up to 12 months in RRMS and up to 4 months in SPMS patients.

**Results:** NAT cessation was associated with a return of disease activity in 38% of the RRMS and 33% of the SPMS patients within 12 and 7 months, respectively. Concentration of free and cell bound NAT as well as α4-integrin-receptor saturation decreased in the RRMS and SPMS patients whereas α4-integrin expression increased over time. NAT induced increase of lymphocytes and its subsets normalized and a non-significant drop of NK and Th17 T-cells counts could be detected. All RRMS patients showed physiological sNfL levels <8pg/ml 1 month after last NAT infusion. During follow-up period sNfL levels peaked up to 16-fold and were linked to return of disease activity in 19 of the 37 RRMS patients. Treatment holiday was also associated with a return of disease activity in 4 of 5 patients and with an increase of sNfL at an individual level.

**Conclusions:** We demonstrate the reversibility of NAT pharmacodynamic and -kinetic markers. sNfL levels are associated with the recurrence of disease activity and can also serve as an early marker to predict present before onset of clinical or radiological disease activity on the individual level.

## Introduction

The recombinant humanized monoclonal antibody natalizumab (NAT) is one of the most effective treatments for relapsing remitting multiple sclerosis (RRMS). NAT binds to the α4 subunit of the α4β1-integrin on circulating mononuclear cells, thus limiting the entry of lymphocytes through the blood brain barrier (1). This mechanism of action impacts the central nervous system immunosurveillance, which is responsible for the development of progressive multifocal leukoencephalopathy (PML), a rare but potentially fatal brain infection caused by the John Cunningham Virus (JCV). A treatment duration of more than 2 years, JCV antibody seropositivity and the use of an immunosuppressive treatment before initiation of NAT therapy have been identified as risk factors for developing a PML (2). In case of treatment discontinuation due to increased PML risk, patients are faced with the possible recurrence or even rebound of disease activity even when switching to another disease modifying therapy (DMT) (3–6). High disease activity and a high level of disability prior to NAT therapy were identified as risk factors for reactivation of clinical disease activity after NAT withdrawal (7). Controlled treatment holidays, different dosing regimens as well as extended interval dosing (EID) were proposed as strategies to reduce PML risk while maintaining efficacy of NAT therapy (8–13). However, efficacy of EID vs. standard interval dosing (SID) is still going to be evaluated in a randomized controlled clinical trial (NCT03689972).

After NAT withdrawal the reversibility of NAT effects on immune cells seems to be linked with the recurrence of disease activity. Whereas, absolute lymphocyte counts increased during NAT therapy due to NAT's mode of action, a decrease of T helper (Th) 17 cells, CD4+ and CD8+ T-cells, CD19+ B-cells and CD56+ NK-cells was observed after NAT cessation (14–17). Regarding pharmacokinetics of NAT, Plavina et al. demonstrated a decrease of free NAT concentration and α4-integrin (CD49d) saturation and an increase of α4-integrin expression levels during washout period, which is in line with our previously published data (17, 18). Lohmann et al. revealed that the extent of NAT induced reduction on CD49d levels but not the kinetics of recovery might predict stable disease course during switching to another treatment (19).

The most promising biomarker of neuroaxonal injury as well as disease activity in multiple sclerosis (MS) is serum neurofilament light chain (sNfL) (20–27). Recently, it has been postulated that sNfL may also serve as a treatment response marker (28, 29). Additionally, sNfL levels have been found to be elevated early during NAT-associated PML and correlate with PML lesion volume (30, 31). Until now, data about sNfL dynamics during NAT washout and under subsequent treatment are missing.

In this study, we address the pharmacokinetics and -dynamics (PK, PD) in association to clinical and subclinical parameters during the washout period of NAT in RRMS and SPMS patients. We aim to identify immunological and serological biomarkers that could assist in individualized management of treatment switch after NAT treatment.

## Methods

### Subjects

In our study, we included at least 64 MS patients on NAT treatment. Different approaches were chosen to answer our study questions: (1) 50 RRMS patients were evaluated that stopped NAT treatment primarily due to increased PML risk and switched to other DMTs (cohort 1). (2) Nine patients with SPMS were included that participated in the phase III study ASCEND and stopped NAT therapy (cohort 2) (32). (3) A third cohort of 5 patients with RRMS was evaluated for both effects of cessation and restart of NAT treatment (treatment holiday, cohort 3). All patients of cohort 3 participated in the phase II, randomized, placebo controlled RESTORE study observing disease activity in MS during a 24-week interruption of NAT therapy (33). Patient characteristics are reported in Table 1.

**Table 1:** Patient characteristics (*N* = 64).

	**Cohort 1**	**Cohort 2**	**Cohort 3**
Patients, n	50	9	5
Disease course	relapsing remitting	secondary progressive	relapsing remitting
Gender, female, n (%)	31 (62)	4 (44)	2 (40)
Age, years, Mean ± SD	39.6 ± 12.1	46.1 ± 8	28.2 ± 8.9
Range	21–62	34–57	20–40
Disease duration, years, Mean ± SD	8.7 ± 5.7	11.1 ± 5.2	6.6 ± 4.1
Range	1–28	4–18	3–11
EDSS, Mean ± SD	3.2 ± 1.7	5.6 ± 0.98	n.a.
Median (range)	3.0 (1.0–8.0)	6.0 (4.0–6.5)	
Previous use DMT, n (%)	33^a^ (73)	5^b^ (100)	5 (100)
Total number of NAT infusions, mean ± SD	37 ± 21	7 ± 3	39 ± 8
Range	6–104	3–10	2–13
Positive JCV antibody status, *n* (%)	45 (90)	7 (78)	n.a.

*Baseline characteristics of evaluated patients. DMT, disease modifying treatment; EDSS, expanded disability status scale*.

a *Data available for 45 of 50 patients*,

b *Data available for five of nine patients*.

All patients were closely screened for the occurrence of clinical confirmed relapses and radiological disease activity, defined by new/enlarging and/or gadolinium enhancing (GdE) lesions in MRI scan. Clinical visits were performed every 4 weeks and patients were screened for relapses by a trained and experienced neurologist. Relapses were defined as new/worsening of neurologic symptoms persisting ≥ 24 h in the absence of fever or infection. MRI was performed at different timepoints within the first 12 months after discontinuation of NAT therapy in cohort 1. Patients in cohort 2 were screened for radiological disease activity with MRI as earliest as 3 weeks after cessation, after 4 and 7–8 months. Patients of cohort 3 were monitored with MRI every 4 weeks.

Blood samples for PK and PD evaluations were obtained every 4 weeks after NAT cessation up to 12 weeks in cohort 1 and up to 16 weeks in cohort 2 and 3. Additional blood samples were collected for up to 20 weeks after restarting NAT treatment in cohort 3.

### Ethical Approval

The immunological substudy was performed according to the Declaration of Helsinki and the study protocol was approved by the Ethics Committee of the Faculty of Medicine of the Dresden University of Technology, Germany. All participants provided written informed consent.

### Immune Cell Phenotyping Using Fluorescence Activated Cell Sorting (FACS)

After blood collection absolute cell counts of T-cells, B-cells and natural killer (NK) cells were measured at the Institute of Clinical Chemistry and Laboratory Medicine, University Hospital in Dresden, Germany. The institute complies with standards required by DIN-EN-ISO 15189:2014 for medical laboratories. Cells were characterized by surface staining with fluorescence labeled anti-CD3, anti-CD4, anti-CD8, anti CD-16, anti CD-14, anti CD-19 and anti CD-56 antibodies (BD Biosciences, San Jose, CA, USA) according to the manufacturer's instructions. Negative controls included directly labeled or unlabeled isotype-matched irrelevant antibodies (BD Biosciences, San Jose, CA, USA). Cell subsets were measured using FACS Canto II flow cytometer (BD Bioscience, San Jose, CA, USA).

For further evaluation of immune cell subsets, peripheral blood mononuclear cells were isolated from the heparinised blood samples using Biocoll separating solution (Biochrom Ag, Berlin, Germany) and Ficoll-Paque (Amersham Biosciences, Amersham, United Kingdom) in LeucoSep tubes (Greiner Bio One, Frickenhausen, Germany). Subpopulations of T-cells were characterized by surface staining with fluorescence labeled anti-FoxP3 and intracellular staining with fluorescence labeled anti-Il17 antibodies (BD Bioscience, San Jose, CA, USA) according to the manufacturer's instructions. Cell frequencies were evaluated on LSR Fortessa cytometer (BD Bioscience, San Jose, CA, USA).

### Measurement of Pharmacodynamic and –Kinetic Data Using a HL60 Cell Based FACS Assay

For analysis of cell bound NAT, CD49d expression and α4-integrin receptor saturation on CD3+ T-cells peripheral blood mononuclear cells were isolated from the heparinised blood samples using Biocoll separating solution (Biochrom Ag, Berlin, Germany) and Ficoll-Paque (Amersham Biosciences, Amersham, United Kingdom) in LeucoSep tubes (Greiner Bio One, Frickenhausen, Germany). Cells were stained with fluorescence-labeled anti-CD3 (BD Bioscience, San Jose, USA), anti-immunoglobulin (IG)-G4 (Southern Biotech, Birmingham, AL, USA), and anti-CD49d (BD Biosciences, San Jose, CA, USA) antibodies, isotype controls were used. Mean Fluorescence intensity (MFI) was analyzed using fluorescence activated cell sorting (FACS, FACS Calibur, BD Bioscience, San Jose, CA, USA). Plasma supernatants were collected and stored at −20°C for subsequent NAT concentration measurements which was performed using our previously described HL60 cell based FACS assay (18).

### Evaluation of sNfL Dynamic Using Single Molecule Analysis (SIMOA)

Serum samples were stored at −80°C until after preparation. sNfL levels were determined using a Simoa HD-1 instrument (Quanterix, Lexington, MA, USA) (23, 34). The Advantage NF-Light singleplex Kit was used and samples were prepared as defined in the manufacturer's instructions (Quartered, Lexington, MA, USA). Sample dilution was calculated and done by the instrument. The mean intra-assay coefficient of variation of duplicates was below 10%.

### Statistical Analysis

Data are expressed as mean ± standard deviation (SD). Our longitudinal patient data were analyzed per cohort by generalized linear mixed models for repeated measures with gamma distribution and log link function due to right-skewed distribution pattern of the data and timepoint as the fixed effect of the model. Bonferroni correction for pairwise tests was used. Values of ^*^p < 0.05, ^**^p < 0.01, ^***^p < 0.001, and ^****^p < 0.0001 were considered as statistically significant. Clinical parameters are depicted in a Kaplan-Meier survival curve for relapses and new and/or GdE lesions in MRI scan. Statistical analyses were performed using the IBM SPSS Software for Windows (Version 25.0; IBM Corporation, Armonk, NY, USA).

## Results

### NAT Cessation in RRMS (Cohort 1) and SPMS (Cohort 2) Patients

#### Clinical and Radiological Data - Cohort 1

At the timepoint of NAT cessation, RRMS patients presented with a mean EDSS about 3.2 ± 1.7 (range 1–8). Patients received a mean number of 37 ± 21 (range 6–104) NAT infusions before treatment stop. Information about pre-treatment was available for 45 of the 50 RRMS patients. 73% of the patients with RRMS had received a DMT before NAT whereas 27% were treatment naïve. The majority of RRMS patients (91.8%) were free of disease activity during NAT treatment. NAT therapy was stopped because of JCV seropositivity (90% positive JCV serostatus), treatment duration over 24 months and/or previous immunosuppressive treatment with increased PML risk in 48 of 50 RRMS patients (Table 1). Only two patients discontinued treatment primarily due to adverse events (one patient with generalized pain, one patient without data available) and one due to pregnancy.

After NAT withdrawal, 37/50 of RRMS patients switched to fingolimod within 3.4 ± 1.1 months and 10/50 to alemtuzumab within 4.6 ± 2.9 months. Overall, the washout period between switching from NAT to another DMT was on average 3.7 ± 1.7 months (range 2–13 months). Two patients received no further treatment due to conversion to SPMS and one patient received no further treatment due to pregnancy.

**Figure 1:**
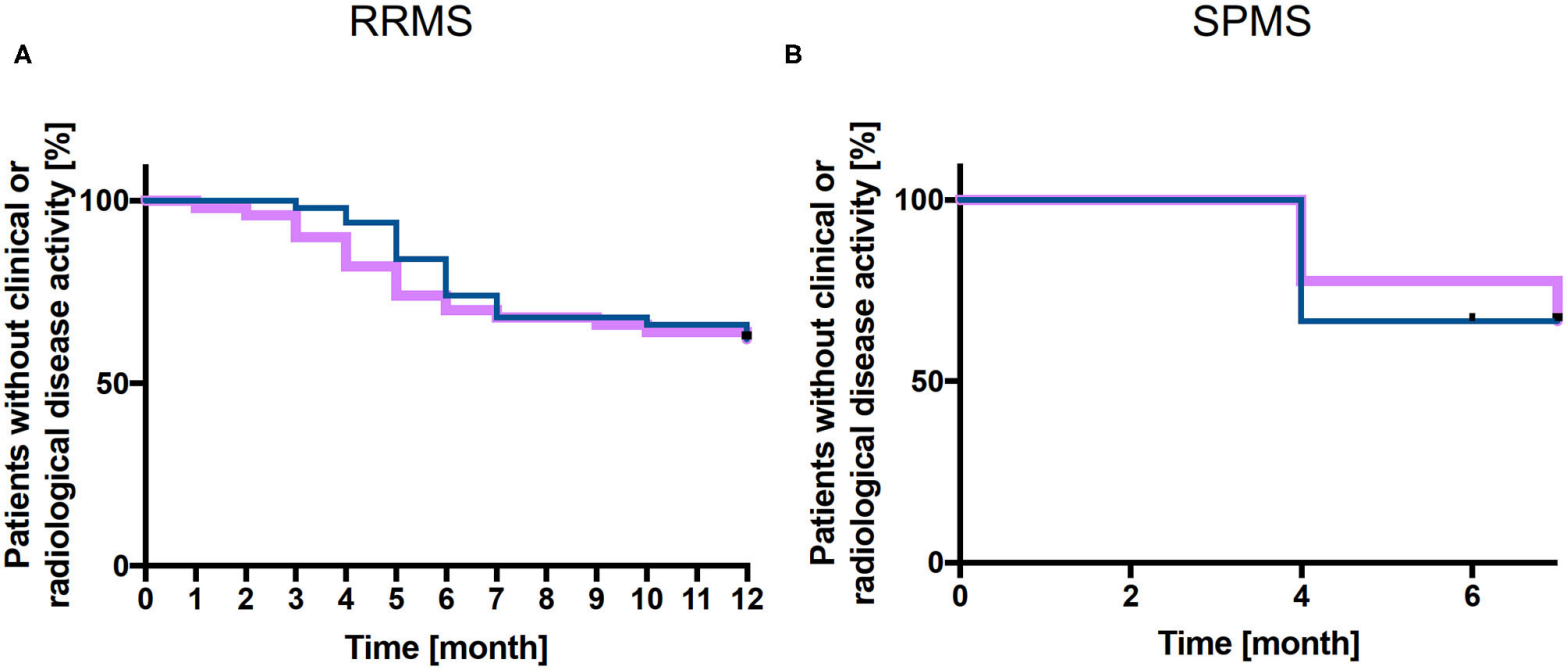
Kaplan-Meier analysis of relapse-free survival and without new and/or enlarging T2 lesions. Relapse free (purple line) and without new/enlarging T2 lesions survival (blue line) is presented for cohort 1 (RRMS patients, *n* = 50) up to 12 months **(A)** and for cohort 2 (SPMS patients, *n* = 9) up to 7 months **(B)** after NAT withdrawal.

The relapse-free survival rate was 70 % at 6 months and 62% at 12 months and survival rate without new/enlarging and/or GdE lesions was 74 % at 6 months and 62% at 12 months in RRMS patients (Figure 1A). Mean time to relapse was 5.2 ± 2.8 months and new/enlarging and/or GdE T2 lesions were revealed within 6 ± 2.2 months. The mean number of new cerebral T2 lesions was 2.2 ± 1.8 in RRMS patients, in 87.5 % of patients new T2 lesions or GdE were detected. A total of 10 out of 50 RRMS patients experienced a relapse and 10 patients presented radiological disease activity while 9 patients suffered both clinical and radiological disease activity. In 6/10 patients presenting with a relapse a new DMT was already started whereas this was the case in 7/10 patients with new/enlarging T2 lesions in cerebral MRI scan. In the 9 patients with both clinical and radiological disease activity, 6 had already started a new DMT before disease activity occurred.

#### Clinical and Radiological Data - Cohort 2

SPMS Patients presented with a mean EDSS about 5.6 ± 1.0 (range 4–6.5) and had received a mean number of 7 ± 3 (range 3–10) NAT infusions. For 5 of the nine SPMS patients information about pre-treatment was available, all of them received a DTM in their previous disease course. The majority of these patients was relapse free during NAT treatment (Table 1). About 78% of patients presented a positive anti-JCV serostatus (Table 1).

After NAT discontinuation, survival relapse free as well as survival without new/enlarging T2 and/or GdE lesions was 67% at 7 months (Figure 1B). In 2 SPMS patients, one resp. 2 new GdE lesions were detected whereas one patient presented with 9 new GdE lesions. Taking into account the clinical and radiological disease activity after NAT withdrawal in these 3 SPMS patients, they received subsequent DMT (1 Rituximab, 2 NAT).

#### Peripheral Immune Cell Subsets

**Figure 2:**
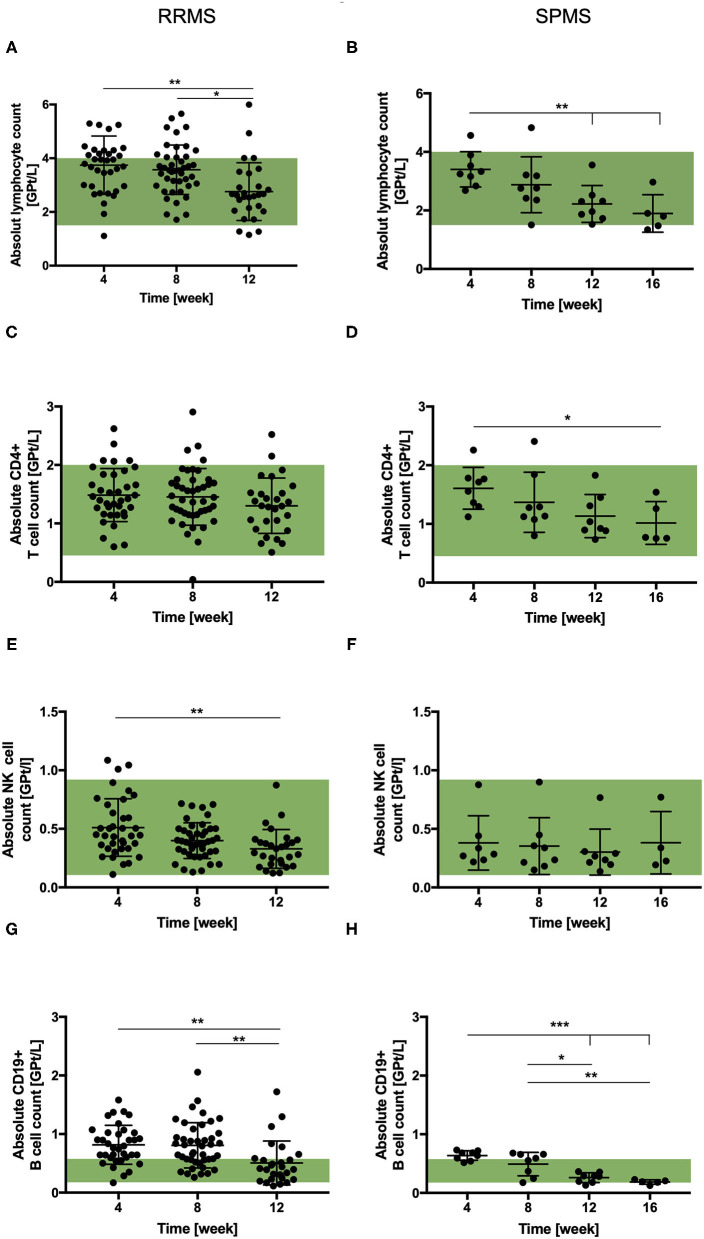
Total lymphocyte and lymphocyte subset count in RRMS (*n* = 50) and SPMS (*n* = 9) patients after the cessation of NAT therapy. Mean absolute cell count ± SD of lymphocytes **(A,B)**, CD4+ T-cells **(C,D)**, NK cells **(E,F)** and CD19+ B-cells **(G,H)** are presented for RRMS (left) up to 12 weeks and for SPMS (right) patients up to 16 weeks after the cessation of NAT treatment. Reference range is green. Data were analyzed by generalized linear mixed models for repeated measures. Asterisks indicate a statistically significant difference (*p < 0.05, **p < 0.01, ***p < 0.001).

For both cohorts, a reduction in absolute lymphocyte count was observed after cessation of NAT therapy. The decrease reached statistical significance at week 12 in both patient cohorts (Figures 2A,B). On average the absolute lymphocyte count remained within the normal range at all timepoints. Cell counts of CD4+ T-cells were not affected by NAT cessation in RRMS within first 12 weeks. A decrease of CD4+ T-cells was documented 16 weeks after NAT stop in SPMS patients (Figures 2C,D).

Frequencies of Foxp3 + Treg-cells were not affected by NAT withdrawal in both cohorts. Cell counts of Th17-cells decreased after the cessation without reaching statistical significance. The NK-cell count tended to decrease after NAT cessation in both patient cohorts, although it did not reached statistical significance in cohort 2 (Figures 2E,F). Absolute cell counts of NKT-cells were not affected by NAT discontinuation. The absolute B-cell count was found to be upper the normal limit in both patient cohorts 4 weeks after NAT withdrawal. After cessation, a decrease was observed in both cohorts reaching statistical significance after week 12 (Figures 2G,H).

#### Plasma NAT Levels

**Figure 3:**
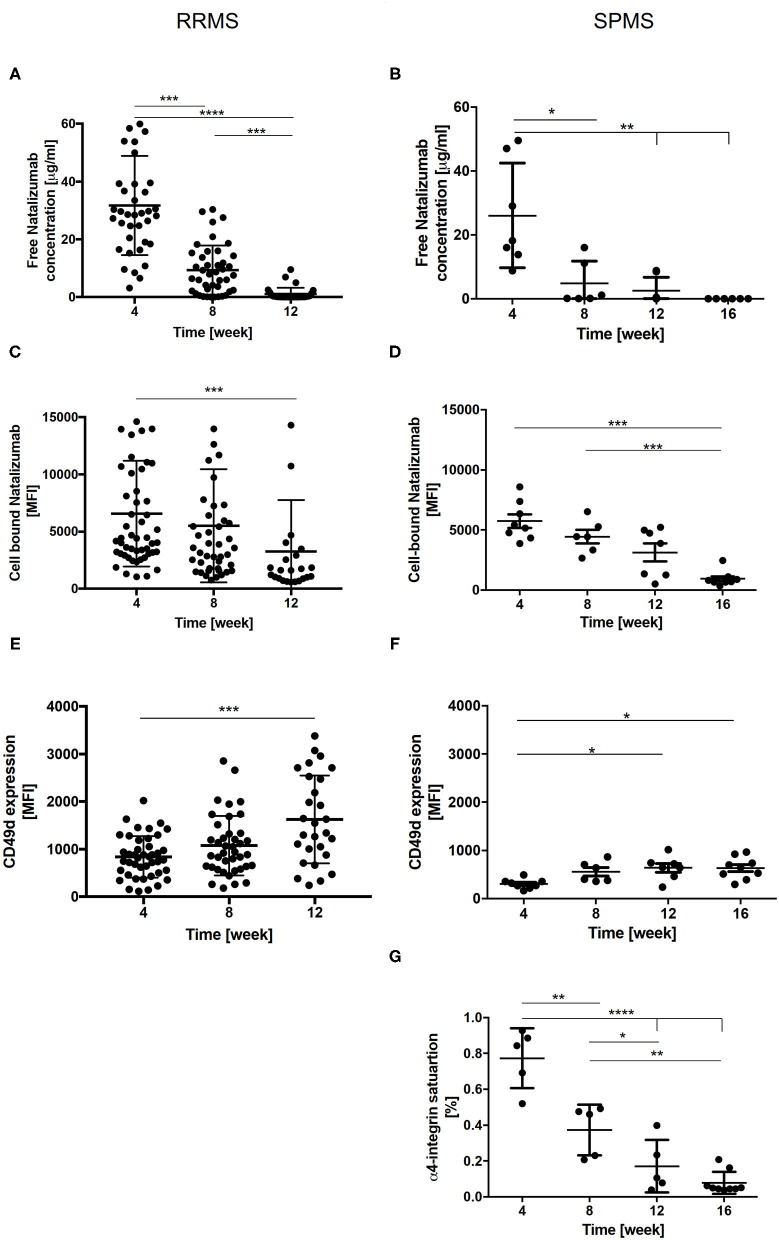
Free NAT concentration, cell bound NAT, CD49d expression and α4-integrin saturation in RRMS (*n* = 50) and SPMS (*n* = 9) patients after the cessation of NAT therapy. Mean values ± SD of free NAT concentration in plasma **(A,B)**, cell bound NAT on CD3+ T-cells **(C,D)** and CD49d expression on CD3+ T-cells **(E,F)** were assessed during the washout of NAT up to 12 weeks in RRMS (left) and up to 16 weeks in SPMS (right) patients. For SPMS patients the mean α4-integrin saturation level on CD3+ T-cells after the cessation of NAT treatment is presented **(G)**. Data were analyzed by generalized linear mixed models for repeated measures. Asterisks indicate a statistically significant difference (*p < 0.05, **p < 0.01, ***p < 0.001, ****p < 0.0001). MFI, mean fluorescence intensity.

The mean free NAT plasma concentration observed 4 weeks after the last infusion was similar in cohort 1 and 2 (33.3 ± 17.5 μg/ml vs. 33.7 ± 9.4 μg/ml). At week 8, NAT concentration levels were significantly decreased in both patient cohorts. Twelve weeks after therapy cessation, NAT concentration was below 2.5 μg/ml or undetectable in the majority of the patients and after 16 weeks, no free NAT was detectable in any patient (Figures 3A,B).

#### Cell Bound NAT

For cell bound NAT on CD3+ T-cells, a mean MFI of 7,486 ± 731 in cohort 1 and of 5,729 ± 1,601 in cohort 2 was detected 4 weeks after last NAT infusion. A significant decrease was revealed 12 weeks after NAT withdrawal in cohort 1 and after 16 weeks in cohort 2 (Figures 3C,D).

#### CD49d Expression and Saturation

A mean CD49d expression on CD3+ T-cells of 819 ± 65 MFI and of 305 ± 100 MFI was observed in cohort 1 and 2 four weeks after NAT cessation. An increase of CD49d expression at week 12 was detected for RRMS and SPMS patients, respectively (Figures 3E,F). In addition, CD49d saturation was analyzed in SPMS patients. The mean CD49d saturation on CD3+ T-cells was 77 ± 7.5% 4 weeks after the last NAT infusion. At week 16 CD49d saturation was decreased to 10% on CD3 + T-cells (Figure 3G). Mean CD49d expression measured 4 weeks after last NAT infusion tended to be higher in RRMS patients with clinical and/or radiological disease activity or with an increase of sNfL as compared to patients without any evidence of disease activity after NAT withdrawal without reaching statistical significance.

#### Serum NfL Levels

A mean sNfL level of 4.6 pg/ml ± 1.7 (IQR 1.5-11 pg/ml) was measured in 46 of the 50 RRMS patients (cohort 1) 4 weeks after NAT cessation. sNfL levels remained stable in the majority of patients within the first 8 weeks of the NAT washout period. During follow up of 12 months there was an increase up to 16-fold sNfL baseline level (range 5.2–101.0 pg/ml) in 37 of 46 patients. The earliest sNfL increase was seen 8 weeks after stopping NAT in 2 RRMS patients, respectively, after 12 weeks in 5 RRMS patients. To evaluate association of sNfL increase with disease activity, a steady state (SS) value of sNfL was defined for the measurement 4 weeks after NAT cessation. A relevant sNfL peak was defined as sNfL value ≥ SS + 2SD. A relevant sNfL peak was documented in 37 RRMS patients 8 weeks after NAT stop. Registered sNfL peaks were associated with clinical and/or radiological disease activity in 19 of the 37 patients. For 11 of this 19 patients an increase of individual sNfL levels, defined as sNfL value ≥ SS + 1 or 2SD was detected 3 (*n* = 1), 2 (*n* = 4) or 1(*n* = 6) month's before first symptoms of relapse appeared and/or MRI activity was detected. For 3 patients with onset of clinical or radiological disease activity the following month, no increase of sNfL levels was detectable the month before. For 5/19 patients no serum sample for sNfL evaluation was available the month before disease activity occurred.

**Figure 4:**
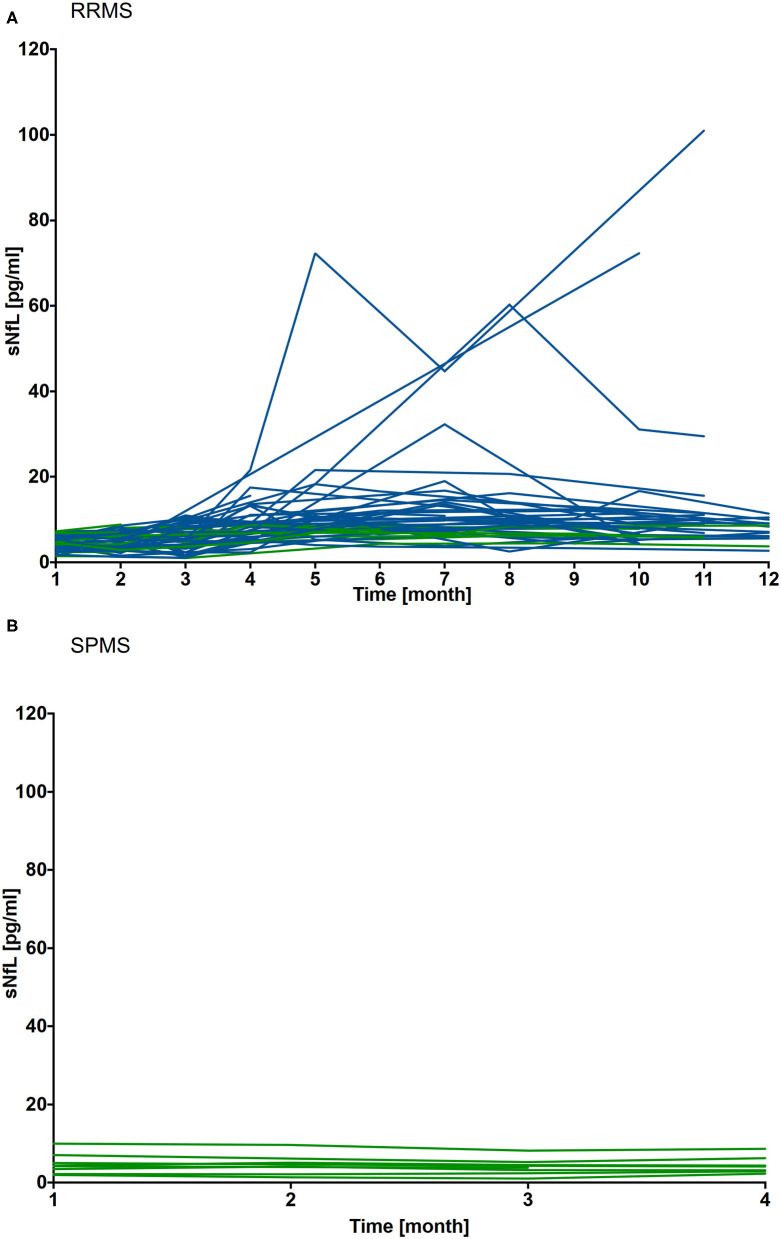
Individual sNfL levels after cessation of NAT treatment in RRMS (*n* = 46) and SPMS (*n* = 9) patients. sNfL levels were assessed during washout period up to 12 months in RRMS patients **(A)** and for 4 months in SPMS patients **(B)**. sNfL value measured 4 weeks after NAT cessation was defined as individual steady state (SS) value. A relevant increase of sNfL was defined as sNfL values ≥ SS + 2SD. Individual sNfL courses are depicted, relevant sNfL increase are labeled blue, patients without an increase are depicted green.

For 18 of the 37 patients, neither new nor worsening symptoms were documented and follow-up MRI showed no new/enlarging T2 or GdE lesions. At the timepoint of sNfL peak, 26 of the 37 patients had already started a new DMT for at least 1 month (Figure 4A).

In SPMS patients (cohort 2), sNfL levels were at 4.8 ± 2.7 (IQR 1.8 – 7.4 pg/ml) 4 weeks after NAT and remained stable at the individual level during the 16 weeks follow up period (Figure 4B).

### NAT Treatment Holiday in RRMS Patients (Cohort 3)

#### Clinical and Radiological Data

**Figure 5:**
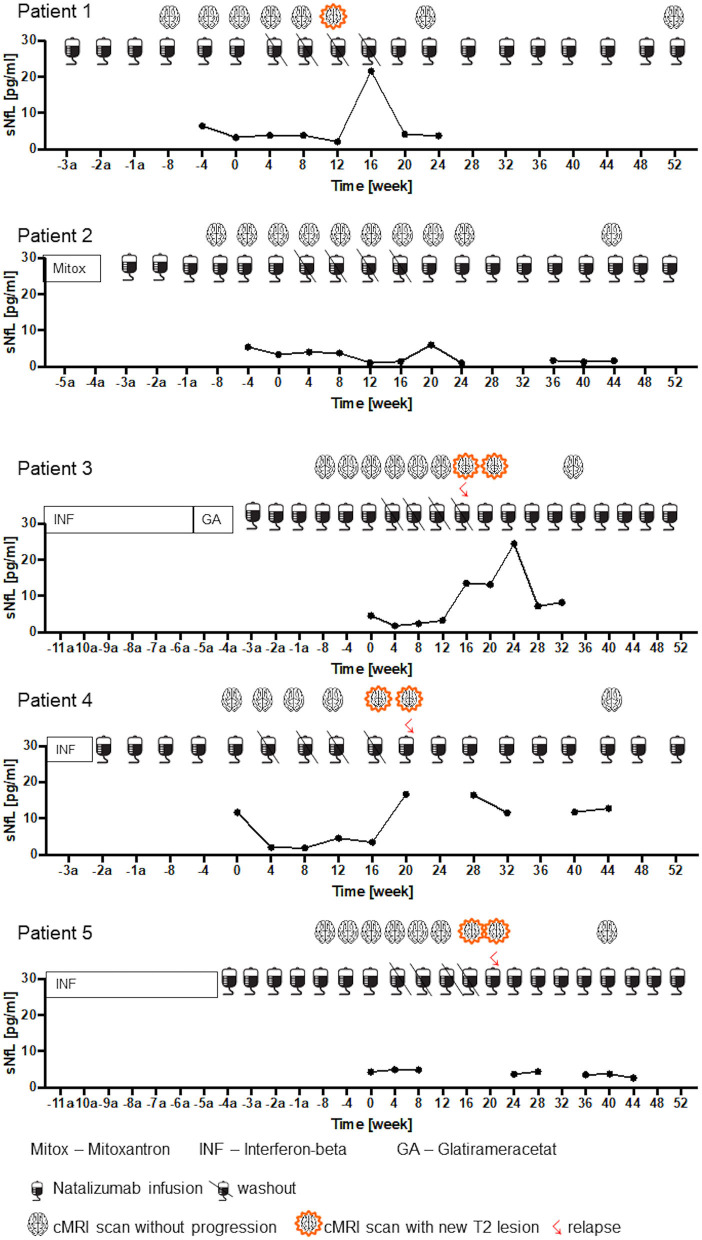
Clinical and radiological disease activity and sNfL dynamics during NAT treatment interruption. sNfL levels during NAT treatment, during washout period and after restart of NAT infusions are presented (*n* = 5). Pre-treatment, timepoint since NAT therapy initiation and clinical confirmed relapses and radiological disease activity are shown up to week 52 = End of study.

In cohort 3, RRMS patients that stopped and restarted NAT were evaluated. A mean number of 39 ± 8 (range 27–48) NAT infusions were administered before patients entered the drug holiday (Table 1). None of the 5 patients experienced clinical disease activity while on NAT treatment. Individual disease course during drug holiday and after re-starting NAT is depicted (Figure 5 patient 1–5). Four of 5 patients presented with new relapses within 20 ± 3.3 weeks after NAT cessation. For all patients with clinical disease activity, radiological disease activity was detected as well. Median time to recurrence of radiological disease activity was 18 ± 2.3 weeks and a median number of 2.5 ± 0.6 new T2 lesions were found. After restart of NAT therapy, regression of demyelinating lesions was documented for 3 of the 5 patients whereas three new lesions without GdE were detected in one patient. All 3 patients were free from clinical disease activity until end of the study (week 52) after re-initiating NAT treatment.

#### Plasma NAT Levels

**Figure 6:**
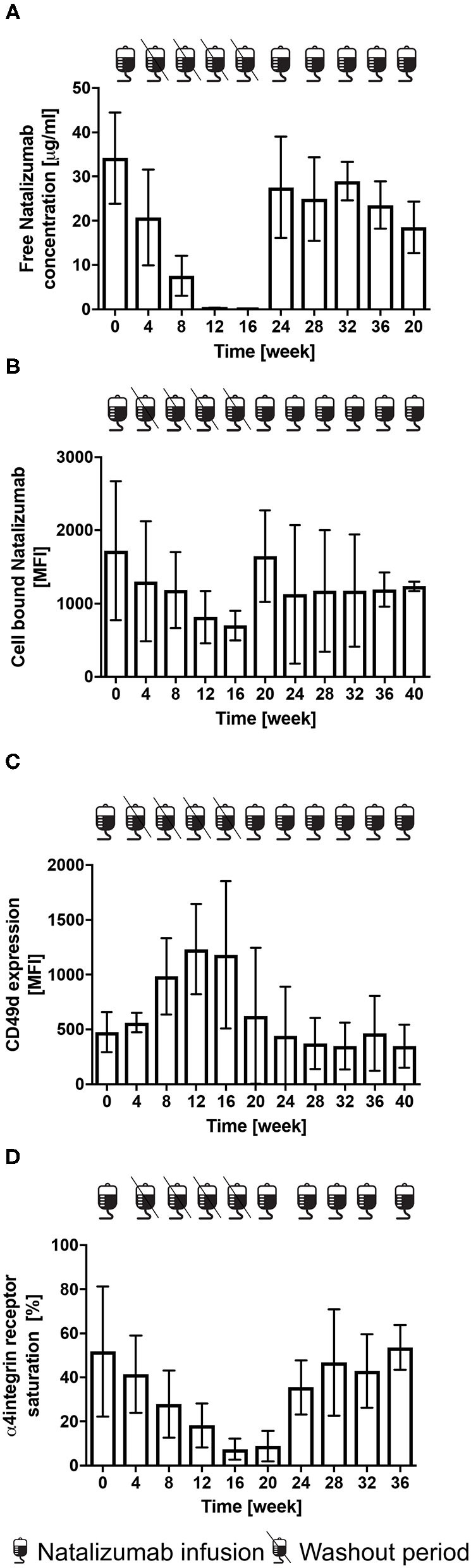
Free NAT concentration, cell bound NAT, CD49 d expression and α4-integrin saturation after the cessation and restart of NAT therapy in RRMS patients (*n* = 5). Mean levels ± SD of free NAT concentration in plasma **(A)**, cell bound NAT on CD3+ T-cells **(B)**, CD49d expression **(C)** and α4-integrin saturation **(D)** on CD3+ T-cells are depicted for baseline = week 0, during washout period and after restart of NAT therapy up to week 40. Data were analyzed by generalized linear mixed models for repeated measures. MFI, mean fluorescence intensity.

A mean free NAT plasma concentration of 34.2 ± 10.3 μg/ml was measured at month 0. During treatment holiday, a decrease of free NAT plasma concentration was observed. The first significant reduction was presented 8 weeks after last infusion (7.6 ± 2.0 μg/ml, *p* < 0.001). Low free NAT plasma concentrations were detectable in all of the 5 patients 16 weeks after NAT interruption. After restart of NAT therapy, a mean free NAT plasma concentration of 18.5 ± 5.8 μg/ml and 27.5 ± 11.5 μg/ml was measured after the first and second infusion, respectively (Figure 6A).

#### Cell Bound NAT

A mean MFI of cell bound NAT on CD3+ T-cells of 1,720 ± 949 was detected at month 0. Compared to the rapid decrease of free NAT concentration, the decrease of cell bound NAT was much slower, the first significant decrease was observed at week 16 (*p* < 0.05). A mean MFI of cell bound NAT on T-cells of 1,647 ± 625 and of 1,127 ± 945 was measured after the first and second infusion after restart (Figure 6B).

#### CD49d Expression and Saturation

Mean CD49d expression on CD3+ T-cells was 477 ± 184 at month 0. After cessation a 2.5-fold increase after 16 weeks was detected. Mean CD49d expression of CD3+ T-cells was 625 ± 620 after the first and 443 ± 446 after the second NAT infusion after the restart (Figure 6C). At month 0, a mean CD49d saturation on CD3+ T-cells of 51.8 % was determined. At week 16 CD49d expression was decreased by 85.7 % (*p* < 0.05). CD49d saturation on CD3 + T-cells was lowest after restart with a mean of 8.8 ± 6.9 %, whereas a mean of 53.6 ± 10.2 was reached after 5 NAT infusions (Figure 6D).

#### Serum NfL Levels

Individual sNfL variation is depicted for each patient in Figure 5. Four patients presented sNfL values below 5 pg/ml at month 0, one patient with a sNfL value of 11.7 pg/ml before drug holiday (Figure 5. patient 4). During NAT treatment stop, an increase up to 24.4 pg/ml was seen in association to clinical confirmed relapse and/or new T2 lesions. After NAT re-initiation, sNfL decreased again in accordance with the stable disease course during the follow up period (Figure 5. patient 1-5).

## Discussion

The monoclonal antibody NAT is one of the most efficacious treatment options for patients with active RRMS. NAT is generally well tolerated, but has the highest risk for PML development among all approved MS treatments. In patients at high risk for developing PML, NAT discontinuation is frequently performed. However, NAT withdrawal remains challenging because it is associated with the recurrence or even rebound of disease activity as demonstrated by several studies and by a recent review of Prosperini et al. (3–6, 12, 33, 35–43). Even the length of NAT washout period and its association to disease activity reactivation remains a point of controversial discussions. A short washout period may decrease the risk of post NAT disease re-activation, but may also increase the risk for carry-over PML: a PML that develops few months after cessation of NAT therapy and after initiating an alternative DMT (44–47).

Indeed, consensus is still lacking in regard to DMT sequencing following NAT withdrawal. Studies addressing treatment switch from NAT to another DMT revealed a superiority of rituximab and alemtuzumab vs. fingolimod in controlling disease activity (48, 49). Recent studies suggested ocrelizumab as a possible choice to reduce the risk of MS disease activity reactivation in patients previously treated with NAT SID and EID (50). Considering the NAT associated elevation in peripheral total and memory B-cells together with the essential role of B-cells in MS pathogenesis, B-cell depleting agents might be a favorable post NAT DMT choice by effectively reducing this cells (51). However, further evaluations including comparisons between alemtuzumab and B-cell depleting therapies with careful observations regarding carry-over PML, are necessary.

Different studies already revealed that the recurrence of disease activity coincides with the decrease of NAT concentration and desaturation of NAT target on the surface of lymphocytes - α4-integrin. However, these markers are not yet well-established in clinical practice although they may be helpful to identify the right individualized timing for the start of an alternative treatment (3, 17). In this study, we assessed clinical and radiological disease activity after the cessation of NAT therapy as well as the reversibility of NAT PK and PD effects in RRMS and SPMS patients.

Clinical and radiological disease activity was detected earliest at 8 weeks after NAT cessation. In our study, 38% of the RRMS patients experienced clinical reactivation of the MS, 83% suffered from a relapse during the first 6 months after NAT withdrawal which confirms data from previous studies in which the proportion of patients with relapses post NAT has ranged from 9 to 80% (43). Even the initiation of a new DMT early after NAT cessation was not able to prevent disease activity following NAT withdrawal, which is in line with a recent published study from Mustonen et al. (7).

The reactivation of disease activity is closely related to reversal NAT effects on PK and PD. Earliest significant changes could be observed 8 and 12 weeks after the last NAT infusion with a decrease of free NAT concentration in plasma and cell bound NAT on CD3+ T-cells, respectively. CD49 expression observed 4 weeks after last NAT infusion tended to be lower in the patients with a stable disease course during follow up as compared to the patients presenting with disease activity. However, statistical significance was not reached. This discrepancy to the results from Lohmann et al. may be influenced by the fact that they compared patients with a stable and an exacerbated disease course defined by a relapse and ≥5 GdE lesions while we compared stable patients and patients with any evidence for disease activity (relapse, new/enlarging T2 lesion's and sNfL peak) (19).

NAT treatment has shown to be associated with increased absolute lymphocyte, CD3+ T-cell, CD4+ T-cell, CD8+ T-cell, CD19+ B-cell and NK-cell counts (14). Our findings are in line with a previous study, in which the effects of NAT on peripheral immune cell subsets were also reversible during washout period (17). In our study, we could observe that cessation of NAT has no effects on FoxP3+ T-regulatory cells which was previously discussed by Stenner et al. (52). Another T-cell subtype, the Th17-cell, is considered to be a critical mediator of disease activity in MS (53). Haas et al. monitored Th17-cell frequency in MS patients without, during and after NAT cessation and found increased frequencies in the immunology periphery during long term treatment as well as a decrease after NAT withdrawal. Additionally, they could observe that Th17-cells became almost undetectable in the blood of patients that presented relapses during the washout period (16). We could detect a Th17-cells drop after NAT cessation likewise, however the decrease was not statistically significant.

Although the immunological pattern may help to identify patients prone to develop clinical and/or radiological disease activity, there is a need for more directed biomarkers that could be implemented into clinical practice. Here, we first present data regarding sNfL dynamics after NAT withdrawal and after starting subsequent DMT during an up to 12 month follow up period. As reported by Gunnarson et al. and Kuhle et al., NFL levels in cerebrospinal fluid decreased during NAT treatment (54, 55). We detected low sNfL levels 4 weeks after last NAT infusion followed by sNfL peaks in 80.4% of the RRMS patients. In general, increases in sNfL levels were linked to reactivation of disease activity and seen up to 3 months before onset of disease activity in some patients. Nevertheless, in 18 of the 37 patients sNfL peaked without evidence of relapse disease or MRI activity. However, patients were only monitored by cerebral MRI as spinal cord MRI was not performed regularly. Other events (trauma, stroke, metabolic diseases) which could be associated with sNfL increase were not reported. As postulated in one of our studies investigating sNfL during alemtuzumab therapy, sNfL peaks without evidence of disease activity can indicate subclinical disease activity (29). For 2 patients, the suspicion of a relapse was reported. However, for both of them no significant variations of sNfL were found leading to the assumption that they do not have suffered from a clinical confirmed relapse. So, sNfL may be a potential tool to proof clinical disease activity and reappearance of disease activity in time-period of planed treatment switch.

To date, only limited data on the cessation of NAT therapy in SPMS patients are available (32). Miravalle et al. investigated a 3- to 4- months drug holiday in 24 RRMS and 8 SPMS patients receiving NAT therapy for a period longer than 12 months. No other DMT was administered during drug holiday. Relapses occurred in 25% of the SPMS and in 38% of the RRMS patient group. This period was associated with new MRI disease activity in nearly all patients (40). Our data are in line with these data demonstrating that the cessation of NAT therapy is associated with a recurrence of disease activity in SPMS patients. PK and PD data of SPMS patients are comparable with RRMS patients stopping NAT. For the investigated time course of the α4-integrin receptor desaturation, our findings are comparable to the findings from Derfuss et al. and Plavina et al. (3, 17). Immune cell frequencies in SPMS patients showed similar patterns as RRMS patients. Frequencies of Th17-cells and NK-cell count decreased after the cessation, although statistical significance was not reached.

Mean sNfL in SPMS patients after NAT cessation did not show yet a significant increase. SPMS patients remained stable and presented without any clinical and radiological disease activity within 4 months after NAT stop. During long-term follow up, three patients presented with return of disease activity in the SPMS group. Unfortunately, no blood samples were available to correlate these clinical characteristics with additional sNfL levels beyond 4 months of follow up.

Our data confirm that drug holiday is not well tolerated and that the reversibility of NAT PK and PD effects coincides with a return of clinical and radiological disease activity. In line with previous findings from Fox et al. and Kaufmann et al., relapses occurred as early as 8 weeks and new or enlarging T2 lesions were detected as early as 12 weeks after NAT cessation. (33, 38). According to our observations from cohort 1 and 3, changes in PK and PD markers were observed as early as 8 weeks after interruption of NAT therapy with significantly reduced free plasma NAT concentrations as previously prescribed (17). 16 weeks after last NAT infusion cell bound NAT and CD49d saturation on CD3+ T-cells were also found to be significantly decreased. In this study, we correlated PK and PD parameters with sNfL measurements. We could show that disease activity reactivation is reflected by sNfL increase at an individual level. Furthermore, we could demonstrate that a drop of sNfL after re-initiation of NAT therapy was linked to a lesion and relapse free disease course. After restarting NAT therapy, CD49d receptor saturation on CD3+ T-cells was found to be above 50% even after the first NAT infusions.

Our data demonstrate that cessation and interruption of NAT therapy is associated with a high risk of recurrence of disease activity in both RRMS and SPMS patients. Although there are some limitations in our observations (limited patient number, different protocols for RRMS and SPMS patients), we present stable effects on clinical data, PK, PK and sNfL level within the first three months after stopping NAT. The return of disease activity is linked to the reversibility of NAT effects on PK and PD. Our observational data do not support the concept of drug holidays in patients with active RRMS treated with NAT. In this context, the concept of EID seems to be better in clinical practice (56).

Additionally, our data suggest that monitoring PD and PK parameters and sNfL may provide guidance to identify the optimal time window for switching to other highly efficacious treatments. sNfL has a high potential as a treatment response marker with regard to a subsequent DMT post-NAT. However, to define its role as a marker for upcoming radiological and clinical disease activity further investigations are required. Free NAT concentration may also serve as a basis for EID and could be the marker that is the easiest to establish in clinical pratice besides sNfL.

In conclusion, a combination of PK and PD parameters could contribute to the future development of individualized NAT treatment schedules. sNfL seems to be a promising biomarker to monitor clinical and subclinical disease activity as well as treatment response. Additional data have to be generated to support our findings and to establish these biomarker combination in daily clinical practice.

## Data Availability Statement

The original contributions presented in the study are included in the article/supplementary material, further inquiries can be directed to the corresponding author/s.

## Ethics Statement

The studies involving human participants were reviewed and approved by the Ethics Commitee of the Technical University Dresden. The patients/participants provided their written informed consent to participate in this study. Written informed consent was obtained from the individual's for the publication of any potentially identifiable images or data included in this article.

## Author Contributions

UP, KA, and TZ: study concept and design and drafting of the manuscript. UP: acquisition of data. UP and KA: analysis and interpretation of data. HI: critical revision of the manuscript for important intellectual content. UP: statistical analysis. KA and TZ: study supervision. All authors contributed to the article and approved the submitted version.

## Conflict of Interest

UP received speaker fee from Merck, Biogen and Bayer. UP received additionally personal compensation from Biogen and Roche for consulting service. HI received speaker fee from Roche. KA received personal compensation from Roche, Novartis, Sanofi and Celegene for consulting service. TZ received personal compensation from Biogen Idec, Bayer, Novartis, Sanofi, Teva and Synthon for consulting services. TZ received additional financial support for research activities from Bayer, Biogen Idec, Novartis, Teva and Sanofi Aventis.

## Abbreviations

DMT: disease modifying therapy
EID: extended interval dosing
FACS: fluorescence activated cell sorting
GdE: gadolinium enhancing
IQR: interquartile range
JCV: John Cunningham virus
MFI: Mean fluorescence intensity
NAT: natalizumab
NK cells: natural killer cells
PD: pharmocodynamic
PK: pharmacokinetic
PML: progressive multifocal leukoencephalopathy
RRMS: relapsing remitting multiple sclerosis
SD: standard deviation
SID: standard interval dosing
SIMOA: single molecule array technology
SPMS: secondary progressive multiple sclerosis
SS: steady state
sNfL: serum neurofilament light chain
Th17 cells: T-helper 17 cells.

## References

[1] Stüve O Bennett JL. Pharmacological properties, toxicology and scientific rationale for the use of natalizumab (Tysabri®) in inflammatory diseases. CNS Drug Rev. (2007) 13:79–95. 10.1111/j.1527-3458.2007.00003.x 17461891 PMC6494150

[2] Bloomgren G Richman S Hotermans C Subramanyam M Goelz S Natarajan A. Risk of natalizumab-associated progressive multifocal leukoencephalopathy. N Engl J Med. (2012) 366:1870–80. 10.1056/NEJMoa1107829 22591293

[3] Derfuss T Kovarik JM Kappos L Savelieva M Chhabra R Thakur A. α4-integrin receptor desaturation and disease activity return after natalizumab cessation. Neurol Neuroimmunol Neuro. (2017) 4:e388. 10.1212/NXI.0000000000000388 28856176 PMC5572051

[4] Fiander MDJ Bhan V Stewart SA Parks NE. Clinical course of relapsing remitting multiple sclerosis post-natalizumab. Can J Neurol Sci. (2019) 46:455–8. 10.1017/cjn.2019.42 31113500

[5] Sorensen PS Koch-Henriksen N Petersen T Ravnborg M Oturai A Sellebjerg F. Recurrence or rebound of clinical relapses after discontinuation of natalizumab therapy in highly active MS patients. J Neurol. (2014) 261:1170–7. 10.1007/s00415-014-7325-8 24728334

[6] Weinstock-Guttman B Hagemeier J Kavak KS Saini V Patrick K Ramasamy DP. Randomised natalizumab discontinuation study: taper protocol may prevent disease reactivation. J Neurol Neuro Psychiatry. (2016) 87:937–43. 10.1136/jnnp-2015-312221 26780938

[7] Mustonen T Rauma I Hartikainen P Krüger J Niiranen M Selander T. Risk factors for reactivation of clinical disease activity in multiple sclerosis after natalizumab cessation. Mul Scler Rel Dis. (2020) 38:101498. 10.1016/j.msard.2019.101498 31864192

[8] Zhovtis Ryerson L Frohman TC Foley J Kister I Weinstock-Guttman B Tornatore C. Extended interval dosing of natalizumab in multiple sclerosis. J Neurol Neuro Psychiatry. (2016) 87:885–889. 10.1136/jnnp-2015-312940 26917698

[9] Bomprezzi R Pawate S. Extended interval dosing of natalizumab: a two-center, 7-year experience. Ther Adv Neurol Dis. (2014) 7:227–31. 10.1177/1756285614540224 25342976 PMC4206618

[10] Tanaka M Kinoshita M Foley JF Tanaka K Kira J Carroll WM. Body weight-based natalizumab treatment in adult patients with multiple sclerosis. J Neurol. (2015) 262:781–2. 10.1007/s00415-015-7655-1 25663410

[11] Foley JF Goelz S Hoyt T Christensen A Metzger RR. Evaluation of natalizumab pharmacokinetics and pharmacodynamics with standard and extended interval dosing. Mult Scl Rel Dis. (2019) 31:65–71. 10.1016/j.msard.2019.03.017 30939392

[12] Clerico M De Mercanti SF Signori A Iudicello M Cordioli C Signoriello E. Extending the interval of natalizumab dosing: is efficacy preserved? Neurotherapeutics. (2019) 353:1–8. 10.1007/s13311-019-00776-7 31452081 PMC7007494

[13] van Kempen ZLE Hoogervorst ELJ Wattjes MP Kalkers NF Mostert JP Lissenberg-Witte BI. Personalized extended interval dosing of natalizumab in MS: a prospective multicenter trial. Neurology. (2020) 95:e745–54. 10.1212/WNL.0000000000009995 32690785

[14] Kaufmann M Haase R Proschmann U Ziemssen T Akgün K. Real-world lab data in natalizumab treated multiple sclerosis patients up to 6 years long-term follow up. Front Neurol. (2018) 9:577. 10.3389/fneur.2018.01071 30581413 PMC6292961

[15] Stüve O. The effects of natalizumab on the innate and adaptive immune system in the central nervous system. J Neurol Sci. (2008) 274:39–41. 10.1016/j.jns.2008.03.022 18474372

[16] Haas J Schneider K Schwarz A Korporal-Kuhnke M Faller S Glehn von F. Th17 cells: a prognostic marker for MS rebound after natalizumab cessation? Mul Scl J. (2017) 23:114–8. 10.1177/1352458516640609 27003947

[17] Plavina T Muralidharan KK Kuesters G Mikol D Evans K Subramanyam M. Reversibility of the effects of natalizumab on peripheral immune cell dynamics in MS patients. Neurology. (2017) 89:1584–93. 10.1212/WNL.0000000000004485 28916537 PMC5634662

[18] Sehr T Proschmann U Thomas K Marggraf M Straube E Reichmann H. New insights into the pharmacokinetics and pharmacodynamics of natalizumab treatment for patients with multiple sclerosis, obtained from clinical and in vitro studies. J Neuro. (2016) 13:16. 10.1186/s12974-016-0635-2 27349895 PMC4924246

[19] Lohmann L Janoschka C Schulte-Mecklenbeck A Klinsing S Kirstein L Hanning U. Immune cell profiling during switching from natalizumab to fingolimod reveals differential effects on systemic immune-regulatory networks and on trafficking of non-t cell populations into the cerebrospinal fluid—results from the tofingo successor study. Front Immunol. (2018) 9:139. 10.3389/fimmu.2018.01560 30050529 PMC6052886

[20] Arrambide G Espejo C Eixarch H Villar LM Alvarez-Cermeño JC Picón C. Neurofilament light chain level is a weak risk factor for the development of MS. Neurology. (2016) 87:1076–84. 10.1212/WNL.0000000000003085 27521440 PMC5027802

[21] Khalil M Teunissen CE Otto M Piehl F Sormani MP Gattringer T. Neurofilaments as biomarkers in neurological disorders. Nat Rev Neurol. (2018) 14:577–89. 10.1038/s41582-018-0058-z 30171200

[22] Barro C Benkert P Disanto G Tsagkas C Amann M Naegelin Y. Serum neurofilament as a predictor of disease worsening and brain and spinal cord atrophy in multiple sclerosis. Brain. (2018) 141:2382–91. 10.1093/brain/awy154 29860296

[23] Disanto G Barro C Benkert P Naegelin Y Schädelin S Giardiello A. Serum neurofilament light: a biomarker of neuronal damage in multiple sclerosis. Ann Neurol. (2017) 81:857–70. 10.1002/ana.24954 28512753 PMC5519945

[24] Kuhle J Barro C Disanto G Mathias A Soneson C Bonnier G. Serum neurofilament light chain in early relapsing remitting MS is increased and correlates with CSF levels and with MRI measures of disease severity. Mul Scl J. (2016) 22:1550–9. 10.1177/1352458515623365 26754800

[25] Kuhle J Nourbakhsh B Grant D Morant S Barro C Yaldizli Ö. Serum neurofilament is associated with progression of brain atrophy and disability in early MS. Neurology. (2017) 88:826–31. 10.1212/WNL.0000000000003653 28148632 PMC5331872

[26] Kuhle J Plavina T Barro C Disanto G Sangurdekar D Singh CM. Neurofilament light levels are associated with long-term outcomes in multiple sclerosis:. Mul Scl J. (2019) 26:1691–9. 10.1177/1352458519885613 31680621 PMC7604552

[27] Kuhle J Kropshofer H Haering DA Kundu U Meinert R Barro C. Blood neurofilament light chain as a biomarker of MS disease activity and treatment response. Neurology. (2019) 92:e1007–15. 10.1212/WNL.0000000000007032 30737333 PMC6442011

[28] Delcoigne B Manouchehrinia A Barro C Benkert P Michalak Z Kappos L. Blood neurofilament light levels segregate treatment effects in multiple sclerosis. Neurology. (2020) 94:e1201–12. 10.1212/WNL.0000000000009097 32047070 PMC7387108

[29] Akgün K Kretschmann N Haase R Proschmann U Kitzler HH Reichmann H. Profiling individual clinical responses by high-frequency serum neurofilament assessment in MS. Neurol Neuroimmunol Neuro. (2019) 6:e555. 10.1212/NXI.0000000000000555 31119188 PMC6501638

[30] Dalla GC Martinelli V Moiola L Sangalli F Colombo B Finardi A. Serum neurofilaments increase at progressive multifocal leukoencephalopathy onset in natalizumab-treated multiple sclerosis patients. Ann Neurol. (2019) 85:606–10. 10.1002/ana.25437 30761586

[31] Loonstra FC Verberk IMW Wijburg MT Wattjes MP Teunissen CE van Oosten BW. Serum neurofilaments as candidate biomarkers of natalizumab-associated PML. Mul Scl J. (2019) 25:284. 10.1002/ana.25523 31192473

[32] Kapoor R Ho P-R Campbell N Chang I Deykin A Forrestal F. Effect of natalizumab on disease progression in secondary progressive multiple sclerosis (ASCEND): a phase 3, randomised, double-blind, placebo-controlled trial with an open-label extension. Lancet Neurol. (2018) 17:405–15. 10.1016/S1474-4422(18)30069-3 29545067

[33] Fox RJ Cree BAC De Sèze J Gold R Hartung H-P Jeffery D. MS disease activity in RESTORE a randomized 24-week natalizumab treatment interruption study. Neurology. (2014) 82:1491–8. 10.1212/WNL.0000000000000355 24682966 PMC4011468

[34] Wilson DH Rissin DM Kan CW Fournier DR Piech T Campbell TG. The simoa HD-1 analyzer: a novel fully automated digital immunoassay analyzer with single-molecule sensitivity and multiplexing. J Lab Auto. (2015) 21:533–47. 10.1177/2211068215589580 26077162

[35] Havla J Gerdes LA Meinl I Krumbholz M Faber H Weber F. De-escalation from natalizumab in multiple sclerosis: recurrence of disease activity despite switching to glatiramer acetate. J Neurol. (2011) 258:1665–9. 10.1007/s00415-011-5996-y 21431380

[36] Vellinga MM Castelijns JA Barkhof F Uitdehaag BMJ Polman CH. Postwithdrawal rebound increase in T2 lesional activity in natalizumab-treated MS patients. Neurology. (2008) 70:1150–1. 10.1212/01.wnl.0000265393.03231.e5 17872364

[37] Gueguen A Roux P Deschamps R Moulignier A Bensa C Savatovsky J. Abnormal inflammatory activity returns after natalizumab cessation in multiple sclerosis. J Neurol Neuro Psychiatry. (2014) 85:1038–40. 10.1136/jnnp-2014-307591 24876183

[38] Kaufman M Cree BAC De Sèze J Fox RJ Gold R Hartung H-P. Radiologic MS disease activity during natalizumab treatment interruption: findings from RESTORE. J Neurol. (2014) 262:326–36. 10.1007/s00415-014-7558-6 25381458

[39] Kerbrat A Le Page E Leray E Anani T Coustans M Desormeaux C. Natalizumab and drug holiday in clinical practice: an observational study in very active relapsing remitting multiple sclerosis patients. J Neurol Sci. (2011) 308:98–102. 10.1016/j.jns.2011.05.043 21665227

[40] Miravalle A Jensen R Kinkel RP. Immune reconstitution inflammatory syndrome in patients with multiple sclerosis following cessation of natalizumab therapy. Arch Neurol. (2011) 68:186–91. 10.1001/archneurol.2010.257 20937940

[41] Killestein J Vennegoor A Strijbis EM Seewann A van Oosten BW Uitdehaag BMJ. Natalizumab drug holiday in multiple sclerosis: poorly tolerated. Ann Neurol. (2010) 68:392–5. 10.1002/ana.22074 20661928

[42] Larochelle C Metz I Lécuyer M-A Terouz S Roger M Arbour N. Immunological and pathological characterization of fatal rebound MS activity following natalizumab withdrawal:. Mul Scl J. (2016) 23:72–81. 10.1177/1352458516641775 27037182

[43] Prosperini L Kinkel RP Miravalle AA Pietro Iaffaldano Fantaccini S. Post-natalizumab disease reactivation in multiple sclerosis: systematic review and meta-analysis:. Ther Adv Neurol Dis. (2019) 12:1756286419837809. 10.1177/1756286419837809 30956686 PMC6444403

[44] Putzki N Clifford DB Bischof D Moore A Weinshenker BG Freedman MS. Characteristics of PML Cases in Multiple Sclerosis Patients Switching to Fingolimod From Natalizumab. Bosten, MA: ECTRIMS Online Library. (2014).

[45] Killestein J Vennegoor A van Golde AEL Bourez RLJH Wijlens MLB Wattjes MP. PML-IRIS during fingolimod diagnosed after natalizumab discontinuation. Case Rep Neurol Med. (2014) 2014:1–4. 10.1155/2014/307872 25506447 PMC4258922

[46] Giovannoni G Marta M Davis A Turner B Gnanapavan S Schmierer K. Switching patients at high risk of PML from natalizumab to another disease-modifying therapy. Pract Neurol. (2016) 16:389–93. 10.1136/practneurol-2015-001355 27114560

[47] Hassoun L Eisele J Thomas K Ziemssen T. Hands on alemtuzumab-experience from clinical practice: whom and how to treat. Mul Scl Dem Dis. (2016) 1:10. 10.1186/s40893-016-0011-1

[48] Alping P Frisell T Novakova L Jakobsson PI Salzer J Björck A. Rituximab versus fingolimod after natalizumab in multiple sclerosis patients. Ann Neurol. (2016) 79:950–8. 10.1002/ana.24651 27038238

[49] Pfeuffer S Schmidt R Straeten FA Pul R Kleinschnitz C Wieshuber M. Efficacy and safety of alemtuzumab versus fingolimod in RRMS after natalizumab cessation. J Neurol. (2019) 266:165–73. 10.1007/s00415-018-9117-z 30446966

[50] Mancinelli CR Scarpazza C Cordioli C De Rossi N Rasia S Turrini MV. Switching to ocrelizumab in RRMS patients at risk of PML previously treated with extended interval dosing of natalizumab:. Mul Scl J. (2020) 27:790–4. 10.1177/1352458520946017 32749910

[51] Planas R Jelcić I Schippling S Martin R Sospedra M. Natalizumab treatment perturbs memory- and marginal zone-like B-cell homing in secondary lymphoid organs in multiple sclerosis. Eur J Immunol. (2012) 42:790–8. 10.1002/eji.201142108 22144343

[52] Stenner M-P Waschbisch A Buck D Doerck S Einsele H Toyka KV. Effects of natalizumab treatment on Foxp3+ T regulatory cells. PLoS ONE. (2008) 3:e3319. 10.1371/journal.pone.0003319 18836525 PMC2553177

[53] van Langelaar J van der Vuurst de Vries RM Janssen M Wierenga-Wolf AF Spilt IM Siepman TA. T helper 17.1 cells associate with multiple sclerosis disease activity: perspectives for early intervention. Brain. (2018) 141:1334–49. 10.1093/brain/awy069 29659729

[54] Gunnarsson M Malmeström C Axelsson M Sundström P Dahle C Vrethem M. Axonal damage in relapsing multiple sclerosis is markedly reduced by natalizumab. Ann Neurol. (2011) 69:83–9. 10.1002/ana.22247 21280078

[55] Kuhle J Malmeström C Axelsson M Plattner K Yaldizli Ö Derfuss T. Neurofilament light and heavy subunits compared as therapeutic biomarkers in multiple sclerosis. Acta Neurol Scand. (2013) 128:e33–6. 10.1111/ane.12151 23763388

[56] Yamout BI Sahraian MA Ayoubi NE Tamim H Nicolas J Khoury SJ. Efficacy and safety of natalizumab extended interval dosing. Mul Scl Rel Dis. (2018) 24:113–6. 10.1016/j.msard.2018.06.015 29982107

